# Variability in Macro- and Micronutrients of 15 Commercially Available Microalgae Powders

**DOI:** 10.3390/md19060310

**Published:** 2021-05-27

**Authors:** Fabian Sandgruber, Annekathrin Gielsdorf, Anja C. Baur, Benjamin Schenz, Sandra Marie Müller, Tanja Schwerdtle, Gabriele I. Stangl, Carola Griehl, Stefan Lorkowski, Christine Dawczynski

**Affiliations:** 1 Junior Research Group Nutritional Concepts, Institute of Nutritional Science, Friedrich Schiller University Jena, Dornburger Str. 29, 07743 Jena, Germany; fabianalexander.sandgruber@uni-jena.de (F.S.); benjamin.schenz@web.de (B.S.); 2 Competence Cluster for Nutrition and Cardiovascular Health (nutriCARD) Jena-Halle-Leipzig, Dornburger Str. 25, 07743 Jena, Germany; gabriele.stangl@landw.uni-halle.de (G.I.S.); stefan.lorkowski@uni-jena.de (S.L.); 3 Competence Center Algal Biotechnology, Anhalt University of Applied Science, Bernburger Straße 55, 06366 Köthen, Germany; annekathrin.gielsdorf@hs-anhalt.de (A.G.); carola.griehl@hs-anhalt.de (C.G.); 4 Institute of Agricultural and Nutritional Science, Martin Luther University Halle-Wittenberg, Theodor-Lieser-Str. 11, 06120 Halle, Germany; anja-christina.baur@landw.uni-halle.de; 5 Department of Food Chemistry, Institute of Nutritional Science, University of Potsdam, Arthur-Scheunert-Allee 114–116, 14558 Nuthetal, Germany; sandramarie.mueller@outlook.com (S.M.M.); tanja.schwerdtle@uni-potsdam.de (T.S.); 6 NutriAct-Competence Cluster Nutrition Research, Berlin-Potsdam, Arthur-Scheunert-Allee 114–116, 14558 Nuthetal, Germany; 7 Institute of Nutritional Science, Friedrich Schiller University Jena, Dornburger Str. 25, 07743 Jena, Germany

**Keywords:** microalgae, nutrients, fatty acids, PUFAs, protein, N-factor, vitamin D, vitamin B_12_, minerals, trace elements

## Abstract

The nutrient composition of 15 commercially available microalgae powders of *Arthrospira platensis*, *Chlorella pyrenoidosa* and *vulgaris*, *Dunaliella salina*, *Haematococcus pluvialis*, *Tetraselmis chuii*, and *Aphanizomenon flos-aquae* was analyzed. The *Dunaliella salina* powders were characterized by a high content of carbohydrates, saturated fatty acids (SFAs), omega-6-polyunsaturated fatty acids (n6-PUFAs), heavy metals, and α-tocopherol, whereas the protein amounts, essential amino acids (EAAs), omega-3-PUFAs (n3-PUFAs), vitamins, and minerals were low. In the powder of *Haematococcus pluvialis*, ten times higher amounts of carotenoids compared to all other analyzed powders were determined, yet it was low in vitamins D and E, protein, and EAAs, and the n6/n3-PUFAs ratio was comparably high. Vitamin B_12_, quantified as cobalamin, was below 0.02 mg/100 g dry weight (d.w.) in all studied powders. Based on our analysis, microalgae such as *Aphanizomenon* and *Chlorella* may contribute to an adequate intake of critical nutrients such as protein with a high content of EAAs, dietary fibers, n3-PUFAs, Ca, Fe, Mg, and Zn, as well as vitamin D and E. Yet, the nutritional value of *Aphanizomenon flos-aquae* was slightly decreased by high contents of SFAs. The present data show that microalgae are rich in valuable nutrients, but the macro- and micronutrient profiles differ strongly between and within species.

## 1. Introduction

Microalgae are one of the oldest organisms known by mankind. It is estimated that there are over 50,000 species worldwide, but only about 30,000 species have been named and partially studied [1]. The food industry has discovered microalgae as a valuable microorganism for their purposes. Microalgae such as *Chlorella* species exhibit a high protein content, and almost all essential amino acids (EAAs), thus serving as a potential source of valuable, high-quality protein. Microalgae can additionally be rich in carotenoids [2,3,4], vitamins [5,6], minerals [7], and omega-3(n3)-fatty acids [8]. At present, only ten microalgae species are authorized by the European Commission for human nutrition [9]. *Aphanizomenon flos-aquae*, *Arthrospira platensis*, *Chlorella luteoviridis*, *Chlorella pyrenoidosa*, *Chlorella vulgaris*, *Dunaliella salina*, *Odontella aurita*, and *Tetraselmis chuii* can be ingested in powder, pellet, or tablet form [10]. *Phaeodactylum tricornutum*, *Schizochytrium* species, and *Ulkenia* species can only be consumed in oil form [10]. *Haematococcus pluvialis* can be ingested as an astaxanthin-enriched extract [10]. Data on the complete nutrient profile of these ten EU-authorized microalgae species are not available at present. Most of the existing data were obtained from microalgae cultivated on a laboratory scale, markedly different from industrial cultivation in open ponds and photobioreactors, thus causing possible variations in the nutrient profile. The variability of the nutrient profile of microalgae according to its species and the cultivation conditions, including culture medium, temperature, and light regime, is a well-studied fact [11,12,13]. Thus, the present study aims to evaluate the nutrient profile of fifteen commercially available microalgae powders and compares the determined data with the nutrient table highlighted in the label information. The total fiber content, the amount of protein and fat, selected minerals, trace elements, heavy metals, vitamins, amino acids profiles, and fatty acids composition of these commercially available microalgae powders were evaluated. Additionally, an individual nitrogen factor (N-factor) for each sample based on the determined amino acid profiles was calculated to ensure the correct quantification of the crude and pure protein.

## 2. Results

### 2.1. Amino Acid Analysis

The analysis of amino acid profiles of the microalgae species under consideration revealed that the sulfur-containing amino acid cysteine (Cys) was present in concentrations below the limit of quantification (LOQ) of 0.001%, and was the rarest in all analyzed samples (Table 1). The Cys concentration in the *Arthrospira platensis* species varied between 0.03 and 0.06% dry weight (d.w.), while the Cys concentration in *Dunaliella salina* and *Haematococcus pluvialis* was below the LOQ of 0.001% d.w. and was significantly different to *Arthrospira platensis* (*p* = 0.028). The amino acid methionine was equally scarce with the lowest concentrations in *Dunaliella salina*, *Haematococcus pluvialis* and *Tetraselmis chuii* powders. The second limiting amino acid was tryptophan with concentrations of 0.03% d.w. in *Dunaliella salina* and 0.37 to 0.55% d.w. in *Arthrospira platensis.* The main amino acids in all species were aspartate (Asp) and glutamic acid (Glu), with the highest concentrations in *Arthrospira platensis* recorded at 4.90 and 5.52% d.w. (Table 1). All analyzed samples were rich in alanine (Ala), arginine (Arg), and leucine (Leu, Table 1). *Dunaliella salina* and *Tetraselmis chuii* powders were deficient in concentrations of all analyzed amino acids with values close to or below the LOQ of 0.001%. In both powders, Glu, Leu, and Val were the main amino acids. The *Dunaliella salina*, *Tetraselmis chuii*, and *Haematococcus pluvialis* powders had concentrations of nitrogen below 2% d.w. These were: 1.5 and 1.0% d.w. in the *Dunaliella* species, 1.94% d.w. for *Tetraselmis*, and 1.04% d.w. in *Haematococcus*, respectively. In contrast, the N-content of the other analyzed powders ranged from 8.5 to 10.4%.

### 2.2. Evaluation of Protein Quality

The amino acid score (AAS), also known as the Chemical Score, was established in 1946 by the World Health Organization (WHO) to quantify dietary protein quality. It evaluates the protein quality by comparing its amino acid concentrations with those of a reference protein [14]. The reference protein was chosen according to WHO criteria for adults’ protein requirements [15]. Amino acids (AAs) with concentrations below the LOQ, e.g., in *Dunaliella salina*, were set to an LOQ of 0.001% to calculate the AAS with the highest possible concentration that could have been in the microalgae power. The calculated AAS of the analyzed microalgae powders varied from 6, being the lowest score in *Dunaliella salina*, to 100, being the highest score in *Chlorella pyrenoidosa* No. 3 and *Arthrospira platensis* powder No. 1 (Table 1).

### 2.3. Macronutrients and Further Components

The nitrogen (N) content quantified by the Kjeldahl method ranged from 1.0 to 10.4 g/100 g between the six species studied (Table 2). There was a wide range of variation due to the multiplication of the N content with several N-factors. With a general N-factor of 6.25, the crude protein content ranged from 0.6 to 65.2%. With the same specific N-factor of 4.78 [16] for all powders, the crude protein variation ranged from 0.5 to 49.9% d.w. Using the calculated specific N-factor based on each microalgae powder’s amino acid profile, the crude protein content could be determined more accurately. In this case, the powders’ crude protein content ranged from 0.21 to 49.6% d.w. Pure protein contents varying between 0.1 to 43.4% d.w. were detected (Table 2).

The total fiber content ranged from 6.3 and 38.7% d.w. in the studied microalgae. The highest concentration of 38.7% total fiber was detected in *Arthrospira platensis* powder (Table 2). The concentration of total fat was below 9% d.w. The quantities of total ash ranged from 0.9 to 11.0% d.w. in the analyzed microalgae. Only the *Tetraselmis chuii* powder showed a higher mean of total ash of 17.7% d.w. Digestible carbohydrate content was calculated based on the protein analysis results, dietary fiber, and ash concentrations. This resulted in a wide range, from 8.8 to 86.6% d.w. digestible carbohydrates within the analyzed powders (Table 2).

### 2.4. Fatty Acid Distribution

The fatty acid composition varied significantly (*p* < 0.05) depending on the analyzed microalgae species (Table 3). The dominating fatty acid in *Arthrospira platensis* was C16:0 with 1.28 and 2.08 g/100 g d.w. High concentrations of C16:0 were also observed in *Chlorella pyrenoidosa* No. 2 (1.47 g/100 g d.w.), *Tetraselmis chuii* (1.49 g/100 g d.w.), and *Aphanizomenon flos-aquae* (1.66 g/100 g d.w.). C18:2_n6_ was the second most abundant fatty acid with 0.90 to 1.49 g/100 g in four of five *Arthrospira platensis* powders. C18:3_n6_ was the third most prevalent fatty acid in four *Arthrospira platensis* powders. In one *Arthrospira platensis* powder, the concentration of C18:2_n6_ was lower than that of C18:3_n6_. Nevertheless, *Chlorella* species had, with 1.97 to 3.14 g/100 g, significantly higher amounts (*p* < 0.05) of C18:2_n6_ than *Arthrospira platensis.* The lowest amounts of saturated fatty acids (SFAs) were detected in *Haematococcus pluvialis* (0.23 g/100 g d.w.). The content of the main SFA C16:0 of *Arthrospira platensis* differed significantly from *Chlorella vulgaris* but not *Chlorella pyrenoidosa* with a p-value < 0.05. C18:1_n9_ varied greatly in *Chlorella pyrenoidosa* powder, with concentrations between 0.14 and 0.55 g/100 g d.w. in two powders and the third with up to 3.43 g/100 g d.w. C18:3_n3_ ranged in two *Chlorella pyrenoidosa* powder extracts from 0.87 to 1.25 g/100 g d.w., whereas the third extract contained only 0.036 g/100 g d.w. C18:2_n6_ was the dominating fatty acid in both *Chlorella* species and varied between 1.97 and 3.14 g/100 g d.w. in the five powders. C18:3_n3_ was the main n3-PUFA with up to 1.30 g/100 g d.w. in *Chlorella vulgaris* No.2, 1.25 g/100 g d.w. in *Chlorella pyrenoidosa* No. 1, and 0.97 mg/100 g d.w. in *Tetraselmis chuii.* These powders also contained good n6/n3 ratios, which were also calculated for *Chlorella vulgaris* No. 1. The n6/n3 ratios of *Chlorella pyrenoidosa* No. 1 (2.1) and No. 2 (2.3), *Chlorella vulgaris* No. 1 (5.0) and No. 2 (1.7), *Tetraselmis chuii* (0.4), *Aphanizomenon flos-aquae* (0.3), and *Haematococcus pluvialis* (1.6) were the lowest and therefore also favorable. The eicosapentaenoic acid (EPA) concentration was 0.41 g/100 g d.w. in the *Tetraselmis chuii* powder analyzed. The remaining powders contained less than 0.01 g/100 g d.w. of EPA and docosahexaenoic acid (DHA). In summary, comparably high amounts of C16:0 (> 25%) were observed in *Tetraselmis chuii*, *Aphanizomenon flos-aquae*, and all *Arthrospira platensis*. *Chlorella pyrenoidosa* No. 3, *Tetraselmis chuii*, and all *Dunaliella salina* contained the highest amounts of C18:1_n9_. C18:3_n3_ concentrations higher than 1.0 g/100 g were observed in *Chlorella pyrenoidosa* No. 1 and *Chlorella vulgaris* No. 2. EPA and DHA values were below 0.01 g/100 g in all powders except for *Tetraselmis chuii*, having 0.41 g/100 g EPA. An n6/n3 ratio lower than 2 was calculated for *Chlorella vulgaris* No. 2, *Tetraselmis chuii*, *Aphanizomenon flos-aquae*, and *Haematococcus pluvialis*.

### 2.5. Minerals, Trace Elements, and Heavy Metals

The concentrations of minerals, trace elements, and heavy metals were identified in all but one microalgae powder (*Dunaliella salina*), as its biomass was limited. There were large differences in Ca and Mg concentrations between the different algae species and the same species obtained from different providers (Table 4). The content of Ca in the analyzed *Arthrospira platensis* powders ranged from 4.5 to 4227 mg/100 g d.w., which is lower than the range described by [17] of 220 to 11,930 mg/100 g d.w. The highest Ca concentrations among the other species were detected in *Chlorella pyrenoidosa* No. 1 with up to 556 mg/100 g d.w. Remarkably, only one of the three analyzed *Chlorella pyrenoidosa* powders had such high concentrations, whereas the means of Ca of the other two powders ranged from 101.6 to 148.2 mg/100 g d.w. The highest Mg concentration was detected in a *Chlorella pyrenoidosa* powder with up to 815.1 mg/100 g d.w., followed by an Mg content of 368.1 mg/100 g d.w. in the *Haematococcus pluvialis* powder. Fe had the highest concentration for the analyzed trace elements, followed by Zn and I (Table 4). Mo concentration was higher than Cu followed by I in only one *Arthrospira platensis* powder. The concentration of Se was below the LOQ of 0.1 µg/100 g d.w. in the *Aphanizomenon flos-aquae*, *Tetraselmis chuii*, one *Arthrospira platensis*, and one *Chlorella pyrenoidosa* powders (Table 4).

The content of selected heavy metals was analyzed in 14 of the 15 microalgae. One *Dunaliella salina* powder had limited biomass (Table 4). The highest Cd concentrations were detected in one *Arthrospira platensis* powder with 9.61 µg/100 g d.w. This value, on average, was three times higher than that of the other microalgae powders. The highest Hg concentrations were detected in another *Arthrospira platensis* powder with 4.91 µg/100 g d.w. *Dunaliella salina* powder contained the highest Pb concentrations with 36.7 µg/100 g d.w. (Table 4). The As concentration was below 70 µg/100 g d.w. in the studied powders, except for one *Arthrospira platensis* powder and one *Dunaliella salina* powder, in which concentrations of 799.2 and 213.7 µg/100 g d.w. were found (Table 4).

### 2.6. Analysis of Vitamins

Vitamins D_2_ and D_3_ were analyzed in all fifteen microalgae powders, as shown in Table 5. In the *Tetraselmis chuii*, *Haematococcus pluvialis*, *Dunaliella salina*, and *Arthrospira platensis* powders, the vitamin D_2_ and vitamin D_3_ content was below an LOQ of 0.013 and 0.4 µg/100 g d.w., respectively. Vitamin D_2_ concentrations between 37.3 and 420.6 µg/100 g d.w. were detected only in *Chlorella* species. Low concentrations, between 0.64 and 1.25 µg/100 g d.w., of vitamin D_3_ were found in two *Chlorella* species and the *Aphanizomenon flos-aquae* powders.

β- and γ- tocopherol concentrations were below the LOQ of 0.5 mg/100 g d.w. in all 15 powders. α-tocopherol was the most abundant tocopherol in all microalgae samples. *Dunaliella salina* powders contained by far the highest concentrations of α-tocopherol with 37.5–46.9 mg/100 g d.w., whereas the concentrations in the remaining powders were on average 5 mg/100 g d.w. In the *Haematococcus pluvialis* powder, the α-tocopherol concentrations were below the LOQ of 0.5 mg/100 g d.w., whereas those in the *Arthrospira platensis* samples were close to the LOQ. δ-tocopherol was detected only in the *Aphanizomenon flos-aquae* powder with 1.90 mg/100 g d.w. In the remaining microalgae powders, the δ-tocopherol content was below the LOQ of 0.5 mg/100 g d.w.

Vitamin B_12_, quantified as cobalamin, was below the LOQ of 0.02 mg/100 g d.w. in all studied powders (data not shown).

### 2.7. Total Chlorophylls and Carotenoids

**Figure 1:**
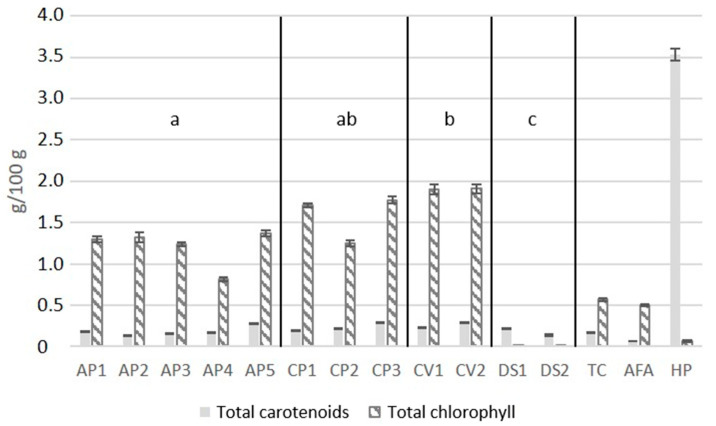
Concentrations of total carotenoids and total chlorophyll in fifteen microalgae in g/100 g d.w. a, b, and c denote significant (*p* < 0.05) differences in chlorophyll concentrations between species. There are no significant differences between the total carotenoid contents of the four species (n = 3; AFA, *Aphanizomenon flos-aquae*; AP, *Arthrospira platensis*; CP, *Chlorella pyrenoidosa*; CV, *Chlorella vulgaris*; DS, *Dunaliella salina*; HP, *Haematococcus pluvialis*; TC, *Tetraselmis chuii*).

*Chlorella vulgaris* powders contained on average the highest chlorophyll concentration at 1.9 g/100 g d.w., followed by *Chlorella pyrenoidosa* at 1.8 g/100 g d.w. (Figure 1). In contrast, the total chlorophyll concentrations in the powders of *Dunaliella salina* and *Haematococcus pluvialis* were less than 0.07 g/100 g d.w. The chlorophyll content of *Dunaliella salina* varied significantly (*p* < 0.05) from *Arthrospira platensis*, *Chlorella vulgaris*, and *Chlorella pyrenoidosa.*

The total carotenoid concentrations ranged from 0.07 in *Aphanizomenon flos-aquae* to 0.29 g/100 g d.w. in *Chlorella vulgaris* (Figure 1). In *Haematococcus pluvialis*, a total carotenoid content of 3.53 g/100 g d.w. was detected, which was more than ten times higher than that of the other analyzed powders (Figure 1). There were no significant differences in total carotenoid content within the four species *Arthrospira platensis*, *Chlorella vulgaris*, *Chlorella pyrenoidosa*, and *Dunaliella salina*.

## 3. Discussion

Microalgae have gained recognition as a viable resource for human nutrition. This study investigated the macro- and micronutrient profiles of fifteen commercially available microalgae powders with seven different species from various companies to evaluate their contribution to nutrient supply for human consumption. In addition, the analyzed amounts of nutrients were compared to those provided by the information label on all acquired products. Here, it has been determined that the amino acid percentages in *Tetraselmis chuii*, *Arthrospira platensis*, and *Chlorella pyrenoidosa* and *vulgaris* were similar to that described in the literature [18,19,20]. Slight differences were identifiable in the amino acid content with functional side-chains. These might occur during the drying process, which was unknown for the studied microalgae powders [21]. Many plants and yeasts can accumulate higher Glu levels due to stress exposure in order to stabilize cell membrane structure and scavenge free radicals [22,23]. The increase in branched amino acids such as Leu and valine (Val) in plants is most likely through abiotic stress caused by nitrogen starvation or high salt content, increasing the carotenoid content in *Dunaliella salina* [24,25]. Furthermore, the quantified nitrogen content is not in line with findings in the literature [26], where it reached 1.6% in *Dunaliella salina* and 4.1–5.2% *in Haematococcus pluvialis* [26]. The calculated AAS shows the high value of protein in *Arthrospira platensis* and *Chlorella vulgaris.* With an AAS of 98 for beef, 121 for eggs, and 127 for milk [27], both microalgae powders are able to compete with animal-based protein. The inconsistencies in the N-factor usage in previous studies have substantially impacted the calculations of the protein content and, therefore, hinder the comparability of the available data on the protein content in microalgae. Only a few research groups published their N-factor used for protein analysis, e.g., for *Arthrospira platensis*, which varied from 4.78 [16,28] to 6.25 [29] and 6.27 [30]. Thus, the differences in the N-factor used can result in under- or overestimation of the calculated protein content. Apart from *Dunaliella salina*, the N-factor of all analyzed microalgae species varied from 4.54 in one *Chlorella vulgaris* powder to 4.90 in *Tetraselmis chuii.* Because the concentration of 14 out of 18 AAs were under the LOQ in *Dunaliella salina* No. 2, it is possible that there is an over- or underestimation of the N-factor calculated for *Dunaliella salina.* Furthermore, a variation in the N-factor with a range of 4.62 to 4.87 was determined for the same *Arthrospira platensis* species but from different dealers. This N-factor coincides with the N-factor of 4.78 used in other studies [16,28].

Due to the multiplication of the N-content with different N-factors, the range of variation in the protein content increased even when the analyzed nitrogen content remained the same. This study used a specific N-factor based on each microalgae powders’ amino acid profile, thus enabling a more accurate calculation of each samples’ crude protein content. What is striking here is the inconsistency in the detected crude protein in *Tetraselmis chuii* with data in the literature [31] that report a crude protein content nearly three times higher. Aside from that, the crude protein content on the information label shows four times higher concentrations than detected. Comparing the crude protein content analyzed with the content labeled on the powder packaging, it is shown that there is an average protein overestimation of 33%, if *Dunaliella salina* and *Tetraselmis chuii* with an overestimation of 3700 and 400% are not included, when using a general N-factor compared to a specific one for each powder. Although the crude protein of *Tetraselmis chuii* does not match the data of the literature, several studies showed the impact of different lighting conditions [12] and availability of micronutrients from the culture medium [11,13] on the protein content of microalgae. Interestingly, the comparison of the crude and pure protein content indicates that *Arthrospira platensis* has the highest concentration of crude protein. However, *Chlorella pyrenoidosa* has the highest concentration of pure protein, caused by a lower non-protein-nitrogen (NPN) content in free amino acids, nucleic acids, chlorophyll [16], or ammonium in all analyzed *Chlorella* powders. Variation in the NPN content could result from different stages of harvesting and medium composition [11].

The concentrations of ash content under 11% and fat under 9%, which were detected in the present study, are comparable with the literature [29]. In comparison to the other analyzed powders, the total ash content in the marine microalgae *Tetraselmis chuii* was 2 to 3 times higher. This might occur due to its cultivation in salt water with higher amounts of minerals and trace elements compared to fresh water. Therefore, the microalgae might accumulate more minerals and trace elements. In addition, the washing process of the biomass, which is generally harder in marine than freshwater microalgae, as well as the chemical dewatering, where inorganic flocculants are used and can contaminate the product with aluminum, iron, and other metals, can affect the amount of ash [32]. The nutrient concentrations analyzed in the present study varies from the labels. Three powders had half the amount of total fat and one double the amount of total fat than that labeled on the packaging. The high content of carbohydrates in *Dunaliella salina* and *Haematococcus pluvialis* and comparably low contents of pure protein and total fiber indicate that these are highly processed microalgae products or that non-standard cultivation conditions have caused the accumulation of other substances [33,34]. Remarkable differences in the total fiber content were seen within powders of the same species. Some *Arthrospira platensis* powders showed 2–3 times higher fiber contents than others. Additionally, none of the determined total fiber contents matched the labeling information, if given. The label information had up to four times lower total fiber concentrations than determined. High intake of dietary fiber is related to reducing total and LDL cholesterol, an improved glucose metabolism, and a longer feeling of satiety [35,36]. Therefore, the nutritional value of the analyzed powder might be far more valuable than labeled on the packaging. 

Microalgae are a known source of long-chain omega-3 polyunsaturated fatty acids (n3-PUFAs), which influence membrane fluidity and function, enzymes, and receptors [37]. As precursors of eicosanoids and docosanoids, long-chain n3-PUFAs influence inflammation processes and immune reactions in humans [38]. The PUFAs content of the microalgae powders was generally high, with 0.7 to 3.9 g/100 g d.w. The most dominant SFA in all powders was C16:0. The amount of C16:0 showed variations throughout all species. Variations of C16:0 were also seen within the same species of *Chlorella* and *Arthrospira*, with up to twice as much C16:0 in the species of *Chlorella.* There is evidence that increased consumption of SFAs such as C16:0 can raise total and LDL cholesterol and can therefore increase the risk for cardiovascular diseases [39]. Oleic acid concentrations (C18:1_n9_) showed considerable variations within the *Chlorella* species with a difference in concentration of up to 26 times. With C18:1_n9_ being the precursor of C18:2_n6_ and C18:3_n3_, *Chlorella pyrenoidosa* 1 and 2 seem to be more efficient in elongation and desaturation into n3-PUFAs by delta-15-desaturase, which may also depend on cultivation [40]. Due to industrialization, the n6/n3 PUFAs ratio typical of the Western diet has shifted from the original ratio of 1:1 to 20:1 [41]. A favorable n6/n3 ratio of 1:1 to 1:5 influences inflammatory processes and plays a role in preventing cardiovascular diseases [39]. Prostaglandin E_2_ and leukotriene B_4_ are two lipid mediators with pro-inflammatory properties [42,43]. Studies showed that the pro-inflammatory properties of prostaglandin E_2_ might exacerbate inflammatory processes though the release of DC-derived IL-23 [44], and therefore, the support of the inflammatory and autoreactive TH17 phenotype in rheumatoid arthritis, as well as bowel diseases [44,45]. Leukotriene B_4_ is an inducer for the activation of neutrophils, monocytes, and eosinophils. Its role in the activation of proinflammatory cytokines and mediators is well studied [46]. Prostaglandin E_2_ and leukotriene B_4_ and can be synthesized in the human body from n6-PUFAs, such as arachidonic acid [46]. Both eicosanoids are vital mediators of thrombosis and inflammation, whereas the resulting eicosanoids from n3-PUFAs have weaker or contrary physiological effects [41]. The regular consumption of the analyzed microalgae such as *Chlorella pyrenoidosa*, *Tetraselmis chuii*, *Haematococcus pluvialis*, and *Aphanizomenon flos-aquae* with n6/n3 ratios between 0.25 and 2.25 could improve the general n6/n3 PUFAs ratio of the modern Western diet. Due to the favorable n6/n3 PUFAs ratio, the *Aphanizomenon flos-aquae* powder seems beneficial for human health. However, its high content of SFAs, in particular, C16:0, reduces its nutritional value. The literature indicates that the content of free fatty acids such as C16:0, C17:1, 18:2n6, and MUFAs stored at room temperature can increase significantly (*p* < 0.05) whilst concentrations of n3-fatty acids and SFAs decrease due to oxidative instability over time [47]. To prevent time-dependent changes in compound compositions due to oxidative stability and photosensitivity, the powders were stored in darkness at −20 °C before analyzing the FAME. In addition, the packaging of the purchased powder was opaque to light and was vacuum-sealed. Nevertheless, it cannot be ruled out that changes of the compound composition happened due to the storage of the powders at the production facility where the storage conditions were mainly unknown.

The problem of high contamination of heavy metals in macroalgae is well documented [48]. Because of their cultivation in open seas, the substances they are exposed to are hardly adjustable, although microalgae contamination is less problematic due to adjustable cultivation media. An impure cultivation medium can still cause contamination. Therefore, the maximum amounts of Cd at < 100 µg/100 g, Hg at < 10 µg/100 g, and Pb at < 300 µg/100 g in microalgae are regulated by the Commission Regulation (EC) No. 1881/2006. None of the 14 analyzed microalgae had amounts that exceeded these limits. There is no regulation by the European Commission for the maximum concentration of As in microalgae or microalgae products. However, the existing guidelines by the Certification of Environmental Standards (CERES) for organic microalgae under the Regulation (EC) 889/2008, NOP, and derived from provisionally tolerable weekly intake (PTWI) for As by the WHO suggest a maximum limit of 70 µg/100 g. Based on these guidelines, *Dunaliella salina* No. 1 and *Arthrospira platensis* powder No. 4 are not suitable for to be declared “organic” (Table 2). By inactivating up to 200 enzymes, mostly involved in cellular energy pathways, DNA synthesis, and repair, As displays its toxicity. Acute As poisoning leads to encephalopathy, peripheral neuropathy, abdominal pain, severe diarrhea, or death [49]. In order to make a clearer statement on the possible negative influence on human health, a compound analysis of As, clarifying if it occurs as an organic or inorganic compound in the powders, was carried out. Inorganic As appears to be toxic, whereas organic As is non-toxic for humans. Nevertheless, all microalgae powders had no concerning concentrations of As for human health [49]. Overall, one microalgae powder of *Arthrospira platensis* (No. 1) had the lowest concentrations (12.91 µg/100 g) of all four determined heavy metals combined. In contrast, the powder of *Arthrospira platensis* (No. 4) showed the highest concentrations of As (799.2 µg/100 g) and Hg (4.91 µg/100 g) and is therefore less suitable for human consumption. These data indicate that the contamination with specific heavy metals does not exclusively depend on the species. Origin and cultivation conditions could be determinants for heavy metal concentrations as the legislation differs between the EU (VO (EU)/2015/2283), the U.S., and Asian countries [50]. Our samples originate mainly from China. In addition, one sample was cultivated in Germany (CP) and one sample was cultivated in Spain (TC; Table 6). The highest concentrations of As (AP, No. 4), Cd (AP, No. 2), Hg (AP, No. 4), and Pb (DS) are detected in samples from China, indicating water contamination or other factors depending on the location of cultivation ponds. This could be attributed to higher limit values constituted in the respective laws which apply for Asian countries. To this day, China has only regulations for heavy metal contents in macro algae but not microalgae [51]. Contained minerals and trace elements were shown in the label information in five out of fifteen powders. Label information of the *Arthrospira platensis* powder No. 1 had 60 to 70 times higher amounts of Ca, Mg, and Fe than detected. Furthermore, the concentrations of minerals and trace elements showed big differences within the same species, especially in the powder of *Arthrospira platensis*, where the amount of Ca was 1000 times higher than the *Arthrospira platensis* powder of a different producer. The Ca concentrations of the powders of *Arthrospira platensis*, *Chlorella pyrenoidosa*, *Chlorella vulgaris*, and *Dunaliella salina* varied significantly (*p* < 0.05) within the same species. These differences might result from different cultivation conditions, particularly the composition and purity of the growing medium. The concentration of the elements also depends on the harvest of the microalgae in different growing stations in which elements were accumulated [52,53]. An additional rinsing of the biomass can also affect the concentrations of minerals in the end product [54]. Again, the consumer will not receive the same product with the same nutrient composition when purchasing powders of the same microalgae species but from different producers. 

The importance of vitamin D for the maintenance of calcium homeostasis in humans is well studied. Thus, an undersupply of vitamin D is associated with an increased risk of osteoporosis, hypertension, diabetes, and autoimmune diseases [55]. The primary source of vitamin D is the synthesis in the skin by photochemical conversion of provitamin D_3_. Apart from that, vitamin D and its precursors are also found, albeit in lower concentrations, in several foods. The highest natural vitamin D source is fish, which accumulates vitamin D_3_ from microalgae, its primary food source [56]. Fungi and yeast contain mainly vitamin D_2_, whereby these foods or food ingredients deliver comparably small concentrations of vitamin D_2_. Microalgae might synthesize vitamin D_3_ by converting 7-dehydrocholesterol due to exposure to ultraviolet B light [57]. Vitamin D_3_ was quantified in the *Aphanizomenon flos-aquae*, *Chlorella vulgaris* (No. 2), and *Chlorella pyrenoidosa* (No. 3) powders. Vitamin D_2_ concentrations were detected in all *Chlorella* powders, with differences of up to 10 times higher amounts in the same species. Within the method mentioned earlier in determining vitamin D_2_ concentration, the impurity of vitamin D_2_ might be from fungi which are present in the cultivation system for symbiotic reasons or due to contamination. Inconsistencies in vitamin D_3_ concentrations are due to the quantification of vitamin D_3_ in only two of five *Chlorella* powders. The α-tocopherol concentrations of *Dunaliella salina* powders were three times higher than the data available from the literature, which might indicate a concentration of α-tocopherol due to the processing of these microalgae [58]. The vitamin B_12_ content was used for advertising *Chlorella pyrenoidosa* (No. 1) with 60 µg/100 g d.w., *Chlorella vulgaris* (No. 2) 300 µg/100 g d.w., and *Arthrospira platensis* (No. 1, 2, 4) 160–350 µg/100 g d.w., even though the concentrations of vitamin B_12_ were below the LOQ of 20 µg/100 g d.w. for all studied microalgae. These data indicate that the advertised concentrations of vitamin B_12_ might be due to the identification of pseudo-cobalamin, whose bioavailability is close to none in humans [59]. Hence, the *Chlorella pyrenoidosa* powder no. 3 is the best vitamin D source, while both *Dunaliella salina* and *Aphanizomenon flos-aquae* powders showed the highest concentrations of α- and δ-tocopherol in all analyzed powders. *Haematococcus pluvialis* and *Arthrospira platensis* powders provide the lowest amounts of vitamins within the analyzed powders.

Chlorophylls are found in photosynthesizing plants, algae, and bacteria and are necessary for harvesting solar energy, charge separation, and electron transport within reaction centers [60]. Because of the free radical scavenging activity of chlorophyll and its derivatives, these substances can act as anti-oxidative and anti-inflammatory agents and are therefore beneficial for human consumption [61]. *Chlorella* is one of the richest sources of chlorophyll [62], which is congruent with the present analysis.

Microalgae can synthesize secondary carotenoids after exposure to environmental stressors such as decreasing nutrients and increasing light intensity [63]. Due to their anti-inflammatory and anti-oxidative properties, carotenoids play an important role in treating age-related macular degeneration [64], the prevention of cardiovascular diseases, different types of cancer [65,66,67,68], and stimulating cell proliferation in bones [69]. *Dunaliella salina* and *Haematococcus pluvialis* are known to accumulate larger concentrations of carotenoids. Under autotrophic conditions, the total carotenoid content in *Haematococcus pluvialis* was up to 1.6 g/100 g d.w. with a total chlorophyll concentration of less than 0.02 g/100 g d.w. [3]. The results of the 14 microalgae differed from the analyzed total carotenoid content of 3.5 g/100 g d.w. in the *Haematococcus pluvialis* powder (Figure 1). Recently published data show that *Haematococcus pluvialis* extracts obtained from the red phase can contain 2.9 g/100 g d.w. total carotenoids [26]. The analyzed chlorophyll and carotenoid concentrations of the studied microalgae were different from those in the literature data. This difference indicates that the purchased *Haematococcus pluvialis* and *Dunaliella salina* powders might be highly processed powders and not the entire algae biomass because there are widely studied taxa for astaxanthin and β-carotene concentrations. In contrast to *Haematococcus pluvialis*, the concentrations of carotenoids in the other microalgae powders were less than 0.3 g/100 g, which is in accordance with the literature. Variations depending on the harvesting times are possible [3,45,57,58]. In addition, the possibility of a decreased carotenoid and chlorophyll content compared to the label information can be caused by their degradation due to a possible exposure to oxygen, heat, acid, and light [70,71,72]. Although attention was paid to vacuum-sealed packaging opaque to light, as well as dark and cooled storage of the powder after arrival, the storage conditions at the production factory were mainly unknown. Therefore, degradation cannot be ruled out.

In summary, a ranking regarding the nutritional value for human nutrition of the analyzed microalgae powder was performed and is shown in Table 7. The order of ranking was performed relating to positive aspects such as the amount of macronutrients, AAS, n3, minerals, and trace elements, as well as vitamins. Less positive components such as low macronutrient contents, SFAs, and high amounts of heavy metals decreased the value of the microalgae powder. *Dunaliella salina* powders were mostly defined by high contents of carbohydrates, Zn, and α-tocopherol. Because of a generally low content of macronutrients such as protein, combined with the lowest AAS and high n6/n3 ratio, *Dunaliella salina* powders were ranked with the lowest nutritional value of all determined powders. The heavy metal analysis of *Dunaliella salina* No.1 revealed high concentrations of As and Pb and was therefore ranked last. Numbers 11 to 13 on the ranking list were given to *Arthrospira platensis* powders No. 3, 2, and 4. All three powders had high amounts of protein and different trace elements compared to ranking numbers 14 and 15. On the negative side, they were low in concentrations of vitamins and high in C16:0 amounts. In addition, the n6/n3 ratio was also high. No.4 had the highest concentration of As of all microalgae powders and was therefore placed on ranking number 13. The *Arthrospira platensis* powder No. 3 had the highest amounts of minerals and Zn, thus it was better ranked than *Arthrospira platensis* powder No. 3. The 10th place was given to the *Haematococcus pluvialis* powder because of the third highest carbohydrate concentration and the by far highest carotenoid amount. The powder had also higher contents of minerals than *Arthrospira platensis* powder No. 2 and the third lowest n6/n3 ratio. Contrariwise, the powder had low concentrations of total fat and protein combined with a low protein quality defined by the AAS. The vitamin concentrations were all under the LOQ. Places 8 and 9 were given to the *Arthrospira platensis* powders No. 1 and 5. The AAS, protein, and fiber content was in both powders generally high. Both powders lacked in vitamins and had a high n6/n3 ratio. Because of a higher n6/n3 ratio and high concentrations of As, the *Arthrospira platensis* powder No. 5 was higher ranked than No. 1, with the lowest heavy metal concentrations. *Chlorella pyrenoidosa* No. 2 had high amounts of total fiber, had the third highest vitamin D_2_ concentrations, and a low n6/n3 ratio. Because of the high concentration of As and low amounts of trace elements, *Chlorella pyrenoidosa* No. 2 was placed at 7. In the powder of *Tetraselmis chuii* the highest EPA concentrations were determined, as well as the second lowest n6/n3 ratio. High amounts of carbohydrates and α-tocopherol additionally defined the nutritional value of *Tetraselmis chuii.* However, the As concentrations were the 4th highest and the chlorophyll amounts the 4th lowest under all microalgae powders. Hence, the powder of *Tetraselmis chuii* was placed at number 6 because of the nutritionally valuable fatty acid profile. The rankings 4 to 5 were given to *Chlorella pyrenoidosa* No. 1 and *Chlorella vulgaris* No. 1. Both powders show their nutritional value throughout high amounts of fiber, vitamin D_2_, and a low n6/n3 ratio. Due to higher contents of minerals and the second lowest amounts of heavy metals, the nutritional value of *Chlorella pyrenoidosa* No. 1 was better ranked than *Chlorella vulgaris* No. 1. The 3rd place was given to *Aphanizomenon flos-aquae* because of the lowest n6/n3 ratio of all microalgae powders and its high concentrations of protein, Fe, Cu, and vitamins. The nutritional value was slightly decreased by its high amounts of C16:0. The powder of *Chlorella vulgaris* No. 2 was ranked 2nd place, because of the second highest AAS and vitamin D_2_ amounts, the highest chlorophyll concentration, 4th lowest n6/n3 ratio, and high protein and fiber contents. *Chlorella pyrenoidosa* No. 3 was named the best microalgae powder of all analyzed products. Although its n6/n3 ratio was slightly increased, the powder had the highest AAS and four times more vitamin D_2_ than the microalgae with the second highest amounts. The powder was additionally rich in protein and total fat. The powder contained overall favorable concentrations of all nutrients valuable for human nutrition.

## 4. Materials and Methods

### 4.1. Microalgae Powders

The following fifteen commercially available microalgae products of different origin were analyzed in this study: *Arthrospira platensis* (*n* = 5), *Chlorella pyrenoidosa* (*n* = 3), *Chlorella vulgaris* (*n* = 2), *Dunaliella salina* (*n* = 2), *Haematococcus pluvialis* (*n* = 1), *Tetraselmis chuii* (*n* = 1), and *Aphanizomenon flos-aquae* (*n* = 1). The microalgae products were purchased as a dried powder from different dealers. Care was taken that the packaging of all purchased powders was opaque to light and the powder was vacuum-sealed. Detailed information on the cultivation conditions, e. g., culture medium, cultivation method, and light-dark cycle, were mostly unknown. Contamination with other microalgae or microorganisms cannot be ruled out. The products were stored in darkness at 7 °C, which was mostly recommended on the label information of the acquired powders. The powders were all purchased in July 2018 and arrived within two weeks. The fifteen microalgae powders were analyzed within 14 days after arrival. The powders were additionally freeze-dried to determine the moisture content. Because the residual moisture of all 15 powders was < 1%, the moisture content was not taken into account for further calculation. For the analysis of the powders, the fresh biomass and not freeze-dried was used to prevent interference of the drying process with the nutrition profile. Aliquots of each microalgae were taken and stored at −20 °C for fatty acid analysis. Table 6 shows the given label information of all fifteen microalgae powders.

### 4.2. Amino Acids and Ammonium Quantification

A total of 20 mg d.w. microalgae powder was subjected to acidic hydrolysis (5 mL 6 N HCl, 48 h, 110 °C, nitrogen as protective gas). In addition, an alkaline hydrolysis (5 mL 4 M NaOH, 24 h, 110 °C, nitrogen as protective gas) was performed for the determination of tryptophan, which is unstable to acidic hydrolysis [73]. Alkaline hydrolysates were acidified with HCl to pH < 2. Acidic and acidified hydrolysates were evaporated, and the residues were equilibrated to the pH of the running program, according to the manufacturer’s instructions, with a Biochrom30+ analyzer (Biochrom, Cambridge, UK) in 5 mL loading buffer (sodium citrate pH 2.2). Norleucine was added as an internal standard. Amino acid separation by pH gradient, post-column ninhydrin derivatization, and detection was carried out by a Biochrom30+ analyzer (Biochrom, Cambridge, UK) to obtain individual amino acid and ammonium content [74]. Buffers and standards were purchased from Laborservice Onken (Gruendau, Germany).

### 4.3. N-Factor Calculation

For the determination of the protein concentration, an N-factor is needed. The N-factor was calculated from the amino acids and other nitrogen-containing molecules and the total organic N content according to the literature [75]. The calculation is seen as follows, with the AA Ala used as an example:

M (Anyhdro Ala) in g/mol:

$$M\left(N\right)\mathrm{in}\frac{g}{\mathrm{mol}}-M\left(H_{2}O\right)\mathrm{in}\frac{g}{\mathrm{mol}}$$

N (Amide) in %:

$$\mathrm{amount}\;\left(\mathrm{Ammonia}\right)\;\mathrm{in}\;\%*\frac{M\left(N\right)\mathrm{in}\;g/\mathrm{mol}}{M\left(\mathrm{Ammonia}\right)\mathrm{in}\;g/\mathrm{mol}}$$

N (Anhydro Ala) in %:

$$\frac{M\left(\mathrm{Ala}\right)\;\mathrm{in}\;g/\mathrm{mol}}{M\left(\mathrm{Anyhdro}\;\mathrm{Ala}\right)\;\mathrm{in}\;g/\mathrm{mol}}*N\left(\mathrm{Ala}\right)\;\mathrm{in}\;\%$$

Amount (Weighted N of Ala) in %:

$$\frac{\mathrm{Amount}\;\left(\mathrm{Ala}\;\mathrm{in}\;\mathrm{protein}\right)\;\mathrm{in}\;\%*N\left(\mathrm{Anhydro}\;\mathrm{Ala}\right)\mathrm{in}\;\%\;}{100}$$

Amount (Anhydro Ala in dry sample) in %:

$$\mathrm{Amount}\;\left(\mathrm{Ala}\;\mathrm{in}\;\mathrm{protein}\right)\;\mathrm{in}\;\%*\;\frac{M\left(\mathrm{Anyhdro}\;\mathrm{Ala}\right)}{M\left(\mathrm{Ala}\right)}$$

Amount (N in Anhydro Ala in dry sample) in %:

$$\mathrm{Amount}\;\left(\mathrm{Anhydro}\;\mathrm{Ala}\right)\;\mathrm{in}\;\%*\;\frac{\mathrm{Amount}\;\left(\mathrm{Anhydro}\;\mathrm{Ala}\;\mathrm{in}\;\mathrm{dry}\;\mathrm{sample}\right)\;\mathrm{in}\;\%}{100}$$

k_A_ (N-factor maximum upper limit):

$$\frac{\mathrm{sum}\left(\mathrm{Amount}\;\mathrm{of}\;\mathrm{Anhydro}\;\mathrm{AAs}\;\mathrm{in}\;\mathrm{dry}\;\mathrm{sample}\right)\;\mathrm{in}\;\%\;}{\mathrm{sum}\left(\mathrm{Amount}\;\mathrm{of}\;N\;\mathrm{in}\;\mathrm{Anhydro}\;\mathrm{AAs}\;\mathrm{in}\;\mathrm{dry}\;\mathrm{sample}\right)\mathrm{in}\;\%+N\;\left(\mathrm{Amide}\right)\;\mathrm{in}\;\%}$$

k_P_ (N-factor maximum lower limit):

$$\frac{\mathrm{sum}\left(\mathrm{Amount}\;\mathrm{of}\;\mathrm{Anhydro}\;\mathrm{AAs}\;\mathrm{in}\;\mathrm{dry}\;\mathrm{sample}\right)\;\mathrm{in}\;\%\;}{\mathrm{amount}\left(N\;\mathrm{in}\;\mathrm{dry}\;\mathrm{sample}\right)\;\mathrm{in}\;\%}$$

N-factor1 (N-factor plausible upper limit):

$$\frac{\mathrm{kA}+\mathrm{kP}}{2}+\frac{\mathrm{kA}-\mathrm{kP}}{4}$$

N-factor2 (N-factor plausible lower limit):

$$\frac{\mathrm{kA}+\mathrm{kP}}{2}-\frac{\mathrm{kA}-\mathrm{kP}}{4}$$

N-factor:

$$\frac{N-\mathrm{factor}1+\;N-\mathrm{factor}2}{2}$$

Table 1 shows the variety of the amino acid profiles within and across the microalgae species, thus warranting the necessity of calculating a specific N-factor for each microalgae powder based on their amino acid profile. For calculating the N-factor, amino acids with a concentration lower than the limit of LOQ of 0.001 g/100 g were calculated as 0.001. 

### 4.4. Quantification of Macronutrients

The N content was quantified using the Kjeldahl method to calculate the pure and crude protein contents according to DIN EN ISO 14891 2002-07. It was multiplied with a general N-factor of 6.25 and a specific N-factor calculated using the amino acid profile and calculation sheet of the National Renewable Energy Laboratory (Golden, CO, USA). The NPN content was calculated using the difference between the crude and pure protein content. The total fiber concentration was enzymatically analyzed with the BIOQUANT^®^ Total Dietary Fiber kit (Merck, Darmstadt, Germany) [76]. The crude ash content was gravimetrically determined after incinerating the microalgae powder in a muffle furnace at 525 °C according to ASU L 06.00-4. The Soxhlet method combined with the acid hydrolysis of Weibull–Stoldt method was used to determine total fat content according to ASU L 06.00-6. The digestible carbohydrate concentration was calculated by subtracting the sum of the individual percentages of ash, total fat, total fiber, and pure protein from 100 g of microalgae biomass.

### 4.5. Lipid Extraction and Fatty Acid Analysis

The total fat in microalgae were extracted using a modification of the Folch/Bligh and Dyer method described by [77]. This extraction uses a methyl-tert-butyl ether instead of chloroform because of less toxicity and harmfulness for humans and the environment. After saponification with NaOCH_3_, the extracted lipids were methylated using BF_3_ as a catalyst for FAME preparation [78]. The FAME analysis of the extract was conducted via gas chromatography (GC; GC-17 V3, Shimadzu, Duisburg, Germany) equipped with an AOC-5000 auto-sampler and flame ionization detector. A fused-silica capillary column DB-225ms (30m × 0.25 mm, i.d. with 0.2 µm film thickness; J and W Scientific, Folsom, USA) with H_2_ as a carrier gas was used. The FAME was measured once. The GC solution software (LabSolution LC/GC release 5.92, Shimadzu, Duisburg, Germany) was used for fatty acid quantification. The FAME amounts were put in relation to the total fat content to determine the fatty acid concentration in the microalgae powders. 

### 4.6. Quantification of Minerals, Trace Elements, and Heavy Metals

Before analyzing the identified minerals, trace elements, and heavy metals, a pressure digestion process with 10% HNO_3_ containing 1 µg/L rhodium in a closed microwave digestion system was applied on all microalgae samples. An inductively coupled plasma mass with a tandem spectrometry (ICP-MS/MS) system (Agilent ICP-QQQ-MS, 8800, Agilent Technologies, Waldbronn, Germany) was used for multi-element detection. A combination of oxygen and hydrogen was used as a reaction/collision gas mixture in mass-shift mode, whereas helium was used as a collision gas in on-mass mode to avoid interference. The Se isotope dilution analysis was carried out with a 10 µg/L solution of enriched ^77^Se and 10 µg/L naturally occurring Se in a 1:1 mixture and in two separate solutions. For guaranteed maximum sensitivity, the nebulizer gas flow as well as the parameters of lenses, Q1, collision cell, and Q2 were tuned daily (oxide ratio < 1.0% (^140^Ce^16^O^+^/^140^Ce^+^), doubly charged ratio < 1.5% (^140^Ce^2+^/^140^Ce^+^), background counts < 0.1 cps). An additional determination of calibration blanks and recalibration checkpoint was performed periodically every 25 samples for quality assurance.

### 4.7. Analysis of Vitamin Content

Microalgae samples were analyzed for vitamin D_2_ and D_3_ content using liquid chromatography–tandem mass spectrometry (LC-MS/MS) and prepared as described by [79]. Before the alkaline saponification, all samples were spiked with deuterated vitamin D_2_ and deuterated vitamin D_3_ (Sigma-Aldrich, Taufkirchen, Germany) as internal standards. Samples were extracted with n-hexane and further purified by normal-phase via high-performance liquid chromatography (HPLC) (1100 Series, Agilent Technologies). All compounds and their corresponding internal standards were identified based on specific retention times and were collected in fractions. These fractions were derivatized with 4-phenyl-1,2,4-triazolin-3,5-dione and injected into the LC-MS/MS (1200 Series, Agilent Technologies; QTRAP 5500, Sciex, Darmstadt, Germany). The conditions of the LC-MS/MS system are described by [80]. Ionization was induced by positive electrospray. Data were recorded in multiple reaction monitoring mode with the depicted ion transitions: vitamin D_2_ 572 > 298, deuterated vitamin D_2_ 575 > 301, vitamin D_3_ 560 > 298, and deuterated vitamin D_3_ 563 > 301.

The concentrations of α-, β-, γ-, and δ-tocopherol were analyzed via HPLC as described by [81]. In brief, 20 mg d.w. of microalgae was saponified with saturated sodium hydroxide and pyrogallol solution (1% in ethanol absolute) and extracted with n-hexane. The tocopherols were separated by HPLC (Agilent 1100 series, Agilent Technologies) with fluorescent detection equipped with a Zorbax Rx-SIL column (250 × 4.6 mm; particle size: 5 µm; Agilent Technologies) [82]. The LOQ for all tocopherols was 0.50 mg/100 g d.w.

Vitamin B_12_ was determined by TeLa (Bremerhaven, Germany) using HPLC-MS/MS and an LOQ of 0.02 mg/100 g d.w.

### 4.8. Total Carotenoids and Chlorophylls

The total carotenoid and chlorophyll content was determined as described by the literature [83,84]. While the total chlorophyll content, defined by chlorophyll a and b, was determined, cyanobacteria such as *Arthrospira platensis* and *Aphanizomenon flos-aquae* only possess chlorophyll a [85,86]. Dry algae biomass was disintegrated using 0.5 mL glass beads (0.75–1 mm) and extracted with ice-cold 90% acetone in a ball mill (Retsch, Haan, Germany) at 30 Hz for 20 min. After adding acetone, the mass was mixed and centrifuged, and after that, the clear supernatant was collected. Extraction was repeated until the biomass was decolorized. The extinction of combined extracts at 450, 647, 664, and 750 nm was measured by photometry (Analytik Jena, Jena, Germany). Total carotenoid and chlorophyll content was calculated as follows and described by the literature [83]:

Carotenoids_total_ (mg/L) = 4.1 ∗ (E_450_ − E_750_) − 0.0535 ∗ Chl_a_ − 0.367 ∗ Chl_b_

Chl_a_ (mg/L) = (11.93 ∗ (E_664_ − E_750_)) − (1.93 ∗ (E_647_ − E_750_))

Chl_b_ (mg/L) = (20.36 ∗ (E_647_)) − (5.5 ∗ (E_664_ − E_750_))

$$\mathrm{Total}\;\mathrm{Carotenoid}/\mathrm{Chlorophyll}\;(\%)=\frac{c\left(\frac{\mathrm{mg}}{L}\right)*V\left(\mathrm{mL}\right)*100\%}{1000*m\left(\mathrm{mg}\right)}$$

Chla_a_ = Concentration of Chlorophyll a
Chl_b_ = Concentration of Chlorophyll b
E = Emission
C = Concentration
V = Volume
m = Mass

### 4.9. Statistical Analysis

Data were expressed as means ± standard deviation. Statistical significance was determined by one-way ANOVA followed by post-hoc analysis (SPSS Statistics 27 Premium). Statistical analysis was performed for microalgae with two or more powders analyzed. In cases where the concentrations were under the LOQ, the value of the LOQ was used to perform statistical analysis. Differences with a *p*-value < 0.05 were considered significant.

## 5. Conclusions

The nutrient profiles of the 15 analyzed microalgae powders were characterized by a high protein, carbohydrate, and dietary fiber content. The use of a general N-factor over a specific factor calculated according to the amino acid profile revealed a slight overestimation of protein and total fiber content. The analyzed contents of nutrients showed strong deviations from the nutrients presented on the label information of each microalgae powder. The variation in the nutrient profile within the same species, but coming from different producers, and the difference to the data in the literature show the dependence of nutrient profiles on the cultivation and processing of the microalgae, presumably for the accumulation of nutrients such as carotenoids. The consumer is not getting the same nutrients of the same microalgae species when purchasing the same microalgae powder from a different producer. Further analysis of the cell structure and detection of contamination due to yeast, bacteria, or other microalgae is necessary to prove the suspicion of the analyzed powders being impure algae biomass or extracts. Thus, most of the analyzed microalgae powders, especially *Aphanizomenon flos-aquae* and *Chlorella* sp., contain various beneficial nutrients such as vitamin D, α-tocopherol, Ca, Fe, Mg, and Zn, and are rich in high-quality protein, n3-PUFAs, and dietary fiber. The nutritional value of *Aphanizomenon flos-aquae*, however, is reduced by its comparably high content of C16:0. In summary, microalgae are a valuable source of various nutrients and are a potential source for human nutrition.

## Figures and Tables

**Table 1:** Amino acid profiles and ammonium contents of 15 microalgae powders in g/100 g d.w. with their calculated protein quality and N-factor.

	*AP*						*CP*				*CV*			*DS*			*TC*	*AFA*	*HP*
	1	2	3	4	5	◊	1	2	3	◊	1	2	◊	1	2	◊	1	1	1
Ala	3.70 ± 0.34	3.65 ± 0.22	3.87 ± 0.10	3.39 ± 0.13	3.77 ± 0.23	a	3.88 ± 0.10	3.440 ± 0.001	3.82 ± 0.11	a	3.81 ± 0.24	3.59 ± 0.19	a	0.009 ± 0.009	<LOQ	b	0.72 ± 0.10	3.66 ± 0.27	0.300 ± 0.001
Arg	3.24 ± 0.35	3.29 ± 0.22	3.45 ± 0.08	3.21 ± 0.12	3.27 ± 0.21	a	2.99 ± 0.06	2.58 ± 0.01	2.87 ± 0.09	b	2.95 ± 0.19	2.69 ± 0.15	b	<LOQ	<LOQ	b	0.52 ± 0.07	2.52 ± 0.19	0.320 ± 0.003
Asp	4.67 ± 0.44	4.70 ± 0.29	4.90 ± 0.13	4.38 ± 0.19	4.70 ± 0.33	a	4.16 ± 0.09	3.76 ± 0.03	4.23 ± 0.15	b	4.06 ± 0.25	3.94 ± 0.21	b	0.010 ± 0.009	<LOQ	c	0.89 ± 0.13	4.41 ± 0.30	0.450 ± 0.003
Cys	0.06 ± 0.01	0.037 ± 0.002	0.071 ± 0.004	0.037 ± 0.003	0.030 ± 0.003	a	0.0486 ± 0.0004	0.023 ± 0.001	0.037 ± 0.004	a	0.046 ± 0.002	0.035 ± 0.003	a	<LOQ	<LOQ	b	0.013 ± 0.002	0.016 ± 0.002	<LOQ
Glu	5.48 ± 0.42	5.40 ± 0.32	5.52 ± 0.16	5.08 ± 0.25	5.44 ± 0.33	a	4.96 ± 0.17	4.46 ± 0.01	4.80 ± 0.11	b	4.78 ± 0.29	4.49 ± 0.23	b	0.027 ± 0.008	0.014 ± 0.001	c	1.22 ± 0.17	5.21 ± 0.36	0.84 ± 0.02
Gly	2.44 ± 0.23	2.41 ± 0.14	2.55 ± 0.07	2.22 ± 0.07	2.49 ± 0.12	a	2.66 ± 0.06	2.25 ± 0.01	2.63 ± 0.07	a	2.55 ± 0.17	2.48 ± 0.14	a	0.010 ± 0.003	0.004 ± 0.004	b	0.58 ± 0.08	2.23 ± 0.18	0.260 ± 0.004
**His**	0.81 ± 0.08	0.80 ± 0.05	0.81 ± 0.02	0.66 ± 0.03	0.79 ± 0.04	a	0.98 ± 0.01	0.84 ± 0.01	0.91 ± 0.03	a	0.94 ± 0.06	0.85 ± 0.05	a	0.011 ± 0.019	<LOQ	b	0.15 ± 0.02	0.76 ± 0.07	0.091 ± 0.004
**Ile**	2.34 ± 0.24	2.32 ± 0.16	2.43 ± 0.03	2.09 ± 0.04	2.36 ± 0.13	a	1.59 ± 0.03	1.336 ± 0.003	1.49 ± 0.02	b	1.46 ± 0.10	1.39 ± 0.08	b	0.004 ± 0.007	<LOQ	c	0.40 ± 0.06	2.13 ± 0.18	0.21 ± 0.03
**Leu**	4.00 ± 0.39	3.98 ± 0.25	4.16 ± 0.10	3.63 ± 0.14	4.04 ± 0.26	a	3.82 ± 0.08	3.38 ± 0.02	3.74 ± 0.11	a	3.70 ± 0.25	3.51 ± 0.19	a	0.027 ± 0.013	0.020 ± 0.019	b	0.84 ± 0.12	3.72 ± 0.27	0.44 ± 0.02
**Lys**	2.23 ± 0.24	2.26 ± 0.14	2.35 ± 0.07	2.07 ± 0.09	2.30 ± 0.16	a	3.81 ± 0.08	2.17 ± 0.01	3.40 ± 0.11	a	3.72 ± 0.23	3.08 ± 0.17	a	<LOQ	<LOQ	b	0.47 ± 0.05	2.42 ± 0.18	0.12 ± 0.01
**Met**	1.09 ± 0.21	0.82 ± 0.08	0.80 ± 0.04	0.65 ± 0.13	0.89 ± 0.14	a	0.73 ± 0.06	0.41 ± 0.07	0.86 ± 0.01	a	0.77 ± 0.11	0.81 ± 0.03	a	<LOQ	<LOQ	b	0.04 ± 0.07	0.43 ± 0.01	0.01 ± 0.02
**Phe**	2.16 ± 0.22	2.16 ± 0.14	2.22 ± 0.05	1.90 ± 0.07	2.16 ± 0.14	a	2.24 ± 0.04	1.97 ± 0.01	2.20 ± 0.06	a	2.17 ± 0.14	2.07 ± 0.12	a	0.006 ± 0.010	<LOQ	b	0.57 ± 0.08	1.96 ± 0.18	0.270 ± 0.004
Pro	2.00 ± 0.17	2.06 ± 0.11	2.10 ± 0.07	1.81 ± 0.05	2.02 ± 0.12	a	2.57 ± 0.18	1.960 ± 0.003	2.41 ± 0.07	a	2.52 ± 0.06	2.30 ± 0.07	a	<LOQ	<LOQ	b	0.59 ± 0.11	1.71 ± 0.08	0.220 ± 0.005
Ser	2.75 ± 0.28	2.76 ± 0.17	2.92 ± 0.07	2.49 ± 0.10	2.80 ± 0.19	a	2.08 ± 0.05	1.89 ± 0.01	2.15 ± 0.07	b	2.07 ± 0.13	2.01 ± 0.11	b	<LOQ	<LOQ	b	0.49 ± 0.07	2.55 ± 0.16	0.280 ± 0.006
**Thr**	2.38 ± 0.25	2.40 ± 0.16	2.50 ± 0.06	2.12 ± 0.09	2.43 ± 0.17	a	2.08 ± 0.05	1.73 ± 0.01	2.17 ± 0.07	a	2.01 ± 0.13	2.05 ± 0.12	a	<LOQ	<LOQ	b	0.49 ± 0.07	2.58 ± 0.18	0.190 ± 0.002
Trp	0.51 ± 0.03	0.37 ± 0.04	0.42 ± 0.16	0.55 ± 0.26	0.41 ± 0.04	a	0.37 ± 0.03	0.31 ± 0.07	0.42 ± 0.05	a	0.33 ± 0.04	0.38 ± 0.05	a	0.028 ± 0.008	<LOQ	b	0.08 ± 0.02	0.39 ± 0.04	0.06 ± 0.02
**Tyr**	2.40 ± 0.27	2.38 ± 0.16	2.50 ± 0.05	2.18 ± 0.09	2.41 ± 0.16	a	2.52 ± 0.07	3.54 ± 0.01	2.54 ± 0.09	a	2.53 ± 0.16	2.27 ± 0.13	a	0.011 ± 0.019	<LOQ	b	0.37 ± 0.05	2.43 ± 0.18	0.46 ± 0.02
**Val**	2.43 ± 0.22	2.41 ± 0.16	2.55 ± 0.05	2.25 ± 0.05	2.48 ± 0.14	a	2.39 ± 0.05	2.10 ± 0.01	2.30 ± 0.05	a	2.23 ± 0.15	2.16 ± 0.13	a	0.024 ± 0.004	0.019 ± 0.003	b	0.55 ± 0.08	2.28 ± 0.19	0.31 ± 0.02
Tau	0.045 ± 0.004	0.034 ± 0.004	0.033 ± 0.003	0.078 ± 0.002	0.0295 ± 0.0005	a	0.05 ± 0.01	0.043 ± 0.001	0.045 ± 0.001	a	0.051 ± 0.003	0.035 ± 0.003	a	0.306 ± 0.037	0.313 ± 0.017	b	0.18 ± 0.03	0.035 ± 0.005	0.301 ± 0.008
MetS	0.16 ± 0.09	0.31 ± 0.05	0.36 ± 0.04	0.34 ± 0.15	0.27 ± 0.14	a	0.23 ± 0.05	0.45 ± 0.05	0.13 ± 0.03	a	0.22 ± 0.06	0.14 ± 0.01	a	<LOQ	<LOQ	b	0.10 ± 0.06	0.33 ± 0.05	0.094 ± 0.003
(Cys)2	0.43 ± 0.04	0.42 ± 0.02	0.42 ± 0.01	0.39 ± 0.02	0.427 ± 0.004	a	0.40 ± 0.02	0.40 ± 0.01	0.451 ± 0.001	a	0.44 ± 0.03	0.43 ± 0.02	a	0.047 ± 0.004	0.053 ± 0.003	b	0.10 ± 0.02	0.29 ± 0.03	0.110 ± 0.001
GABA	0.059 ± 0.002	0.062 ± 0.001	0.059 ± 0.002	0.05 ± 0.01	0.054 ± 0.003	a	0.08 ± 0.01	0.083 ± 0.003	0.071 ± 0.001	a	0.09 ± 0.01	0.063 ± 0.003	a	<LOQ	0.003 ± 0.005	b	0.014 ± 0.002	0.02 ± 0.03	0.021 ± 0.001
Orn	0.027 ± 0.005	0.021 ± 0.005	0.022 ± 0.002	0.021 ± 0.002	0.023 ± 0.001	a	0.025 ± 0.001	0.040 ± 0.002	0.036 ± 0.01	a	0.026 ± 0.001	0.026 ± 0.001	a	<LOQ	<LOQ	a	<LOQ	0.50 ± 0.04	0.028 ± 0.001
NH_4_^+^	0.76 ± 0.10	0.71 ± 0.05	0.75 ± 0.01	0.66 ± 0.02	0.76 ± 0.03	a	0.92 ± 0.02	0.95 ± 0.01	0.80 ± 0.01	b	0.80 ± 0.05	0.79 ± 0.05	b	0.034 ± 0.011	0.033 ± 0.002	c	0.14 ± 0.01	0.86 ± 0.07	0.174 ± 0.008
**Evaluation of protein quality**																			
NEA	23.50 ± 0.85	23.40 ± 0.57	24.44 ± 0.26	21.58 ± 0.37	23.67 ± 0.69	a	22.87 ± 0.30	21.33 ± 0.04	22.61 ± 0.26	a	22.36 ± 0.53	21.13 ± 0.43	a	0.089 ± 0.024	0.024 ± 0.004	b	4.87 ± 0.28	22.21 ± 0.62	2.80 ± 0.03
SEA	4.05 ± 0.35	4.09 ± 0.22	4.26 ± 0.08	3.87 ± 0.12	4.06 ± 0.22	a	3.97 ± 0.06	3.42 ± 0.02	3.78 ± 0.10	a	3.89 ± 0.20	3.54 ± 0.16	a	0.012 ± 0.019	0.002 ± 0.001	b	0.67 ± 0.08	3.28 ± 0.20	0.41 ± 0.01
EAA	17.14 ± 0.69	16.72 ± 0.43	17.43 ± 0.22	15.25 ± 0.36	17.08 ± 0.44	a	17.03 ± 0.16	13.40 ± 0.10	16.59 ± 0.19	a	16.39 ± 0.45	15.45 ± 0.34	a	0.103 ± 0.020	0.045 ± 0.019	b	3.45 ± 0.21	15.92 ± 0.49	1.61 ± 0.05
AAS (%)	100 ± 1.5	94 ± 0.74	94 ± 0.22	71 ± 0.37	94 ± 0.76	a	82 ± 0.3	59 ± 0.2	100 ± 0.3	a	88 ± 0.9	94 ± 0.6	a	6 ± 3.1	13 ± 0.02	b	29 ± 1.4	59 ± 1.1	18 ± 0.8
N-factor	4.87	4.72	4.76	4.75	4.62	a	4.63	4.67	4.73	a	4.67	4.54	a	0.71	0.42	b	4.90	4.63	4.85

AAS, amino acid score; AFA, *Aphanizomenon flos-aquae*; Ala, alanine; AP, *Arthrospira platensis*; Arg, arginine; Asp, aspartic acid; CP, *Chlorella pyrenoidosa*; CV, *Chlorella vulgaris*; Cys, cysteine; DS, *Dunaliella salina*; EAA, essential amino acids; GABA, γ-aminobutyric acid; Glu, glutamic acid; Gly, glycine; His, histidine; HP, *Haematococcus pluvialis*; Ile, isoleucine; Leu, leucine; LOQ, limit of quantification; Met, methionine; MetS, S-adenosylmethionine; NEA, nonessential amino acids; Orn, ornithine; Phe, phenylalanine; Pro, proline; SEA, semi-essential amino acids; Ser, serine; Tau, taurine; TC, *Tetraselmis chuii*; Thr, threonine; Trp, tryptophan; Tyr, Tyrosine; Val, valine; ◊ different letters indicate significant differences between the microalgae species (*p* < 0.05; *n* > 1); LOQ < 0.001; values are expressed as means ± SD (*n* = 3).

**Table 2:** Contents of nitrogenous compounds and main nutrients in 15 microalgae powders in (g/100 g d.w.).

	*AP*						*CP*				*CV*			*DS*			*TC*	*AFA*	*HP*
	1	2	3	4	5	◊	1	2	3	◊	1	2	◊	1	2	◊	1	1	1
**Protein**																			
Nitrogen (Kjeldahl)	9.25 ± 0.05	10.33 ± 0.07	10.0 ± 0.2	10.4 ± 0.1	8.8 ± 0.4	a	9.12 ± 0.07	8.5 ± 0.1	9.83 ± 0.08	a	9.6 ± 0.2	9.6 ± 0.1	a	1.5 ± 0.2	1.0 ± 0.8	b	1.94 ± 0.06	9.3 ± 0.2	1.04 ± 0.04
Crude protein (N-factor 6.25)	57.8 ± 0.3	64.5 ± 0.5	62.7 ± 1.2	65.2 ± 0.7	55.1 ± 2.2	a	57.0 ± 0.4	53.0 ± 0.6	61.4 ± 0.5	a	60.0 ± 1.2	60.1 ± 0.4	a	0.9 ± 0.1	0.6 ± 0.5	b	12.1 ± 0.4	58.4 ± 1.5	6.5 ± 0.3
Crude protein (N-factor 4.78)	44.2 ± 0.2	49.3 ± 0.4	48.0 ± 1.0	49.9 ± 0.6	42.2 ± 1.7	a	43.6 ± 0.3	40.6 ± 0.5	47.0 ± 0.4	a	45.9 ± 1.0	46.0 ± 0.6	a	0.7 ± 0.1	0.5 ± 0.4	b	9.3 ± 0.3	44.6 ± 1.1	5.0 ± 0.2
Crude protein (specific N-factor)	45.1 ± 0.3	48.7 ± 0.4	47.8 ± 1.2	49.6 ± 0.7	40.8 ± 2.0	a	42.4 ± 0.4	39.6 ± 0.6	46.5 ± 0.5	a	44.8 ± 1.1	43.7 ± 0.7	a	0.21 ± 0.01	0.24 ± 0.03	b	9.5 ± 0.4	43.2 ± 1.4	5.1 ± 0.3
Pure protein (specific N-factor)	38.3 ± 1.6	39.7 ± 0.2	39.9 ± 0.4	43.4 ± 1.7	37.8 ± 0.7	a	40.7 ± 0.5	34.0 ± 0.1	41.9 ± 0.3	a	41.0 ± 0.5	39.2 ± 0.3	a	0.12 ± 0.2	0.10 ± 0.1	b	9.1 ± 0.1	33.0 ± 1.4	3.3 ± 0.5
NPN	1.4 ± 0.3	1.90 ± 0.09	4.7 ± 0.2	5.6 ± 0.3	4.2 ± 0.1	a	0.3 ± 0.1	1.2 ± 0.1	1.0 ± 0.1	a	0.8 ± 0.2	1.0 ± 0.1	a	0.09 ± 0.20	0.14 ± 0.10	a	0.08 ± 0.06	2.2 ± 0.4	0.4 ± 0.1
**Further macronutrients**																			
Total fiber	30.9 ± 3.5	13.0 ± 1.1	10.9 ± 1.4	13.2 ± 1.5	38.7 ± 1.5	a	36.6 ± 2.6	21.9 ± 0.2	18.6 ± 1.7	a	29.8 ± 1.4	21.9 ± 1.1	a	6.3 ± 0.1	6.8 ± 0.1	a	9.2 ± 0.3	19.1 ± 2.2	15.8 ± 0.5
Total fat	4.32 ± 0.03	4.8 ± 0.2	4.7 ± 0.2	5.6 ± 0.3	4.2 ± 0.1	a	8.1 ± 0.5	7.7 ± 0.3	8.6 ± 0.2	b	8.4 ± 0.2	8.7 ± 0.2	b	3.0 ± 0.1	3.05 ± 0.05	c	8.0 ± 0.1	4.4 ± 0.1	2.1 ± 0.1
Carbohydrates	15.4 ± 3.9	34.9 ± 1.1	36.9 ± 1.4	31.2 ± 2.3	13.6 ± 1.6	a	8.8 ± 2.7	28.6 ± 0.4	25.1 ± 1.8	a	15.9 ± 1.5	24.9 ± 1.2	a	86.6 ± 0.2	85.8 ± 0.2	b	55.9 ± 0.4	36.6 ± 2.6	77.9 ± 0.8
**Further main components**																			
Ash	11.0 ± 0.2	7.6 ± 0.1	7.62 ± 0.03	6.59 ± 0.03	5.77 ± 0.01	a	5.83 ± 0.02	7.8 ± 0.04	5.70 ± 0.02	a	4.90 ± 0.03	5.329 ± 0.001	a	3.985 ± 0.003	4.24 ± 0.05	a	17.74 ± 0.04	6.9 ± 0.1	0.91 ± 0.03

AFA, *Aphanizomenon flos-aquae*; AP, *Arthrospira platensis*; CP, *Chlorella pyrenoidosa*; CV, *Chlorella vulgaris*; DS, *Dunaliella salina*; HP, *Haematococcus pluvialis*; NPN, non-protein-nitrogen; TC, *Tetraselmis chuii*; ◊ different letters indicate significant differences between the microalgae species (*p* < 0.05; *n* > 1); values are expressed as means ± SD (*n* = 3).

**Table 3:** Fatty acid composition in mg/100 g d.w. of 15 microalgae powders.

Fatty Acids	*AP*						*CP*				*CV*				*DS*		*TC*	*AFA*	*HP*
	1	2	3	4	5	◊	1	2	3	◊	1	2	◊	1	2	◊	1	1	1
***SFAs***																			
C12:0	4.11	6.69	0.46	3.12	4.46	a	1.59	1.08	1.56	a	1.42	2.36	a	8.73	7.65	b	2.49	4.40	0.49
C14:0	65.6	15.7	6.97	14.5	24.2	a	25.3	21.3	25.6	a	21.0	23.4	a	15.8	15.6	a	22.9	275	5.95
C16:0	1278	1895	2076	1790	1809	a	857	1467	1195	ab	813	942	b	776	802	b	1486	1662	177
C18:0	435	97.0	44.8	249	388	ab	288	53.1	855	ab	273	160	a	453	486	b	40.6	75.5	37.6
C20:0	6.55	2.87	2.10	4.69	7.59	a	6.18	2.50	25.0	a	5.46	7.36	a	7.71	9.43	a	2.93	1.51	1.67
C22:0	2.09	0.65	<LOQ	0.73	2.44	a	1.50	3.56	13.9	ab	1.53	5.57	a	8.73	12.9	b	<LOQ	<LOQ	0.65
C24:0	9.57	<LOQ	<LOQ	1.08	4.42	ab	6.61	14.0	35.7	a	5.62	6.59	a	2.54	4.96	b	2.03	<LOQ	1.28
**MUFAs**																			
C14:1_n5_	0.96	0.18	0.41	0.53	0.59	a	0.59	1.09	<LOQ	a	<LOQ	0.80	a	<LOQ	<LOQ	a	<LOQ	17.4	0.28
C16:1_n7_	146	146	152	110	129	a	45.4	33.3	31.5	b	40.3	115	ab	1.86	2.68	c	42.5	122	29.5
C17:1_n7_	3.90	1.20	7.99	1.96	4.71	a	14.9	35.3	9.85	b	18.2	7.01	ab	0.51	0.55	c	2.97	7.56	3.36
C18:1_n9_	134	190	115	136	175	a	137.7	547	3430	ab	158	179	a	777	797	b	1844	136	37.8
C18:1_n7_	31.5	20.8	16.0	23.6	<LOQ	ac	47.5	90.8	26.9	ab	50.5	92.1	b	13.9	14.3	c	173	22.7	24.7
C20:1_n9_	8.07	<LOQ	<LOQ	0.86	4.13	a	3.06	14.06	8.86	a	3.88	2.79	a	3.64	3.94	a	165	0.76	0.63
C22:1_n9_	5.22	1.49	<LOQ	<LOQ	1.81	a	<LOQ	<LOQ	<LOQ	a	<LOQ	<LOQ	a	<LOQ	<LOQ	a	<LOQ	<LOQ	<LOQ
**n6—PUFAs**																			
C18:2_n6_	635	1392	942	896	1489	a	2592	1971	2184	b	3143	2196	b	895	739	a	482	217	512
C18:3_n6_	699	628	781	489	915	a	1.85	18.3	<LOQ	b	<LOQ	2.53	b	0.67	0.30	b	44.6	<LOQ	8.33
C20:2_n6_	12.8	6.59	4.13	5.17	15.2	a	9.17	3.12	5.85	a	9.12	5.70	a	0.17	<LOQ	b	4.20	0.78	1.22
C20:3_n6_	59.5	12.0	5.99	6.45	20.4	a	<LOQ	<LOQ	<LOQ	b	<LOQ	<LOQ	b	<LOQ	<LOQ	b	3.50	<LOQ	<LOQ
C20:4_n6_	37.5	6.08	1.98	3.00	19.2	a	<LOQ	<LOQ	<LOQ	b	<LOQ	1.19	b	<LOQ	<LOQ	b	44.4	2.45	0.29
C22:5_n6_	2.46	0.43	<LOQ	0.74	6.51	a	<LOQ	<LOQ	1.61	a	<LOQ	<LOQ	a	<LOQ	<LOQ	a	<LOQ	<LOQ	<LOQ
**n-3 PUFAs**																			
C18:3_n3_	2.23	1.54	4.61	3.28	4.02	a	1247	868	35.5	b	624	1299	b	1.28	1.40	a	972	880	326
C20:5_n3_	2.52	0.60	4.31	1.40	9.30	a	7.14	8.85	3.55	a	5.95	4.61	a	4.39	6.94	a	406	6.74	1.12
C22:6_n3_	0.54	<LOQ	0.47	<LOQ	2.54	a	<LOQ	<LOQ	<LOQ	a	<LOQ	<LOQ	a	<LOQ	<LOQ	a	<LOQ	<LOQ	<LOQ
**Sum**																			
SFAs	1801	2018	2130	2063	2240	a	1186	1563	2152	a	1121	1147	a	1273	1339	a	1557	2018	225
MUFAs	330	360	292	273	315	a	249	721	3507	ab	271	397	a	797	819	b	2228	306	96.2
PUFAs	1451	2047	1744	1405	2482	a	3857	2869	2230	ab	3782	3510	b	901	748	c	1956	1107	849
Others	739	305	624	409	544	a	3327	2557	191	ab	3516	3326	b	79.1	94.5	c	2300	960	880
n6	1445	2045	1735	1400	2466	a	2603	1992	2191	a	3152	2206	a	895	740	b	578	220	522
n3	5.29	2.14	9.39	4.69	15.9	a	1254	877	39.1	b	630	1304	b	5.67	8.34	a	1378	887	327
n6/n3	273	956	185	299	155	a	2.08	2.27	56.1	b	5.01	1.69	b	158	88.7	a	0.42	0.25	1.59

AFA, *Aphanizomenon flos-aquae*; AP, *Arthrospira platensis*; CP, *Chlorella pyrenoidosa*; CV, *Chlorella vulgaris*; DS, *Dunaliella salina*; HP, *Haematococcus pluvialis*; LOQ, limit of quantification; MUFAs, monounsaturated fatty acids; PUFAs, polyunsaturated fatty acids; SFAs, saturated fatty acids; TC, *Tetraselmis chuii*; LOQ < 0.1 mg/100 g d.w.; ◊ different letters indicate significant differences between the microalgae species (*p* < 0.05; *n* > 1).

**Table 4:** Concentrations of minerals, trace elements, and heavy metals in 14 microalgae powders.

		*AP*						*CP*				*CV*			*DS*	*TC*	*AFA*	*HP*	*LOQ*
		1	2	3	4	5	◊	1	2	3	◊	1	2	◊	1	1	1	1	
**Minerals**																			
Ca	mg/100 g	4.54 ± 0.41	12.5 ± 0.6	4227 ± 128	809.1 ± 31.2	149.1 ± 1.1	a	538.6 ± 25.4	101.6 ± 1.3	148.2 ± 4.6	a	87.1 ± 1.3	112.2 ± 0.7	a	75.7 ± 2.0	184.8 ± 6.2	183.5 ± 8.8	157.2 ± 5.0	0.11
Mg	mg/100 g	4.05 ± 0.05	22.4 ± 1.2	267.8 ± 10.7	228.5 ± 10.6	2304 ± 10	a	815.1 ± 35.0	235.6 ± 3.46	282.4 ± 2.5	b	286.8 ± 1.4	276.3 ± 2.6	b	267.2 ± 1.57	304.6 ± 15.4	294.8 ± 13.6	368.1 ± 13.2	0.48
**Trace elements**																			
Fe	mg/100 g	1.16 ± 0.01	4.96 ± 0.08	6.97 ± 0.10	49.2 ± 1.4	83.5 ± 1.5	a	20.7 ± 0.8	48.1 ± 0.9	21.98 ± 0.04	a	54.0 ± 0.7	53.1 ± 1.2	a	52.7 ± 1.1	51.8 ± 1.9	110.4 ± 2.5	97.2 ± 3.7	0.07
Mn	µg/100 g	<LOQ	<LOQ	3.89 ± 0.11	<LOQ	5.14 ± 0.15	a	9.13 ± 0.52	<LOQ	<LOQ	a	<LOQ	<LOQ	a	<LOQ	5.28 ± 0.25	5.67 ± 0.28	5.25 ± 0.19	3.23
Ni	µg/100 g	8.04 ± 1.92	16.6 ± 0.5	47.5 ± 1.5	72.9 ± 12.1	41.43 ± 0.07	a	61.2 ± 1.7	71.3 ± 3.2	75.4 ± 0.6	b	90.2 ± 1.6	71.5 ± 1.7	b	111.0 ± 7.8	13.23 ± 0.78	20.3 ± 2.6	32.5 ± 1.9	2.45
Cu	µg/100 g	11.0 ± 0.3	188.4 ± 4.6	575.4 ± 0.4	489.4 ± 3.8	300.0 ± 8.3	a	623.0 ± 13.4	56.6 ± 0.2	79.9 ± 1.3	a	65.3 ± 14.1	76.7 ± 5.1	a	182.7 ± 0.2	521.4 ± 3.8	499.4 ± 2.6	274.7 ± 18.3	5.83
Zn	µg/100 g	<LOQ	11943 ± 166	2404 ± 10	351.5 ± 82.6	2144 ± 75	a	1211 ± 120	607.6 ± 4.3	1790 ± 14	a	1032 ± 38	758.0 ± 63	a	2106 ± 21	2073 ± 99	1938 ± 3	1610 ± 54	39.85
Mo	µg/100 g	3.06 ± 0.02	36.9 ± 0.9	66.3 ± 2.0	558.8 ± 1.2	26.2 ± 0.9	a	34.8 ± 0.1	14.4 ± 0.1	11.08 ± 0.07	a	13.07 ± 0.28	11.8 ± 0.7	a	16.30 ± 0.02	50.9 ± 0.1	47.09 ± 0.5	27.7 ± 1.3	0.14
Se	µg/100 g	<LOQ	2.34 ± 0.09	0.30 ± 0.14	6.40 ± 0.04	7.74 ± 0.22	a	<LOQ	7.02 ± 0.65	13.2 ± 2.6	a	8.90 ± 0.47	8.17 ± 0.15	a	9.52 ± 0.44	<LOQ	<LOQ	6.15 ± 1.31	0.20
I	µg/100 g	273.9 ± 204.7	252.2 ± 319.7	431.4 ± 75.3	195.4 ± 214.9	372.8 ± 58.4	a	238.5 ± 140.9	308.1 ± 32.9	280.1 ± 210.8	a	211.9 ± 144.3	207.7 ± 107.1	a	213.9 ± 149.8	245.9 ± 288.4	173.3 ± 230.7	555.2 ± 129.0	0.05
**Heavy metals**																			
As	µg/100 g	0.89 ± 0.14	10.8 ± 0.2	49.4 ± 0.7	799.2 ± 14.8	47.0 ± 1.6	a	5.1 ± 0.1	70.0 ± 0.7	19.4 ± 0.2	a	40.5 ± 2.3	27.9 ± 0.3	a	213.7 ± 3.7	55.1 ± 0.5	60.37 ± 0.05	45.2 ± 1.3	0.03
Cd	µg/100 g	0.54 ± 0.26	9.61 ± 1.56	2.27 ± 1.91	1.96 ± 0.01	1.72 ± 0.50	a	0.46 ± 0.07	1.18 ± 0.26	1.64 ± 0.20	a	0.97 ± 0.02	1.66 ± 0.30	a	2.69 ± 0.84	0.59 ± 0.16	0.58 ± 0.30	1.48 ± 0.01	0.20
Hg	µg/100 g	0.58 ± 0.07	0.76 ± 0.16	0.25 ± 0.07	4.91 ± 1.02	3.96 ± 0.11	a	0.377 ± 0.001	0.59 ± 0.37	0.428 ± 0.001	a	0.55 ± 0.04	0.68 ± 0.07	a	1.51 ± 0.43	0.40 ± 0.14	0.3835 ± 0.0001	3.33 ± 0.64	0.2
Pb	µg/100 g	10.9 ± 7.72	6.8 ± 8.71	22.7 ± 16.06	4.25 ± 5.31	16.48 ± 10.3	a	4.16 ± 5.17	32.0 ± 10.7	31.1 ± 11.4	a	8.92 ± 9.20	30.2 ± 11.7	a	36.7 ± 18.7	5.90 ± 7.64	4.98 ± 6.33	11.2 ± 9.5	0.5

AFA, *Aphanizomenon flos-aquae*; AP, *Arthrospira platensis*; CP, *Chlorella pyrenoidosa*; CV, *Chlorella vulgaris*; DS, *Dunaliella salina*; HP, *Haematococcus pluvialis*; TC, *Tetraselmis chuii*; LOQ, limit of quantification; ◊ different letters indicate significant differences between the microalgae species (*p* < 0.05; *n* > 1); values are expressed as means ± SD (*n* = 2).

**Table 5:** Concentration of studied vitamins and precursors in 15 microalgae powders.

		*AP*						*CP*				*CV*			*DS*			*TC*	*AFA*	*HP*
		1	2	3	4	5	◊	1	2	3	◊	1	2	◊	1	2	◊	1	1	1
**Vitamin D**																				
Vitamin D_2_ *	µg/100 g	<LOQ	<LOQ	<LOQ	<LOQ	<LOQ	a	37.3 ± 2.9	68.7 ± 1.7	420.6 ± 1.7	b	55.4 ± 3.5	110.3 ± 7.7	b	<LOQ	<LOQ	a	<LOQ	<LOQ	<LOQ
Vitamin D_3_^o^	µg/100 g	<LOQ	<LOQ	<LOQ	<LOQ	<LOQ	a	<LOQ	<LOQ	1.25 ± 0.03	a	<LOQ	0.639 ± 0.007	a	<LOQ	<LOQ	a	<LOQ	0.96 ± 0.07	<LOQ
**Tocopherols**																				
α-tocopherol ^#^	mg/100 g	1.20 ± 0.14	<LOQ	<LOQ	<LOQ	0.85 ± 0.40	a	3.62 ± 0.34	3.94 ± 0.55	4.78 ± 0.37	b	3.85 ± 0.23	4.50 ± 0.51	b	46.90 ± 1.96	37.47 ± 1.41	c	9.49 ± 0.07	1.98 ± 0.44	<LOQ
δ-tocopherol ^#^	mg/100 g	<LOQ	<LOQ	<LOQ	<LOQ	<LOQ	a	<LOQ	<LOQ	<LOQ	a	<LOQ	<LOQ	a	<LOQ	<LOQ	a	<LOQ	1.90 ± 0.50	<LOQ

AFA, *Aphanizomenon flos-aquae*; AP, *Arthrospira platensis*; CP, *Chlorella pyrenoidosa*; CV, *Chlorella vulgaris*; DS, *Dunaliella salina*; HP, *Haematococcus pluvialis*; TC, *Tetraselmis chuii*; LOQ, limit of quantification; *, LOQ = 0.013 µg/100 g d.w.; ^o^, LOQ = 0.4 µg/100 g d.w.; #, LOQ = 0.5 mg/ 100 g d.w.; ◊ different letters indicate significant differences between the microalgae species (*p* < 0.05; *n* > 1); values are expressed as means ± SD (*n* = 2).

**Table 6:** Label information of commercially acquired microalgae.

	*AP*					*CP*			*CV*		*DS*		*TC*	*AFA*	*HP*
	1	2	3	4	5	1	2	3	1	2	1	2			
**Energy (kJ)**	1463	1306	1626	1553	1782	/	1221	/	1693	1448	893	/	1421	/	/
**Protein**	60	56.6	62.8	60.40	60	/	60.70	/	55	61.3	7.4	/	38	/	/
**Fat total**	8.2	4.10	6.4	4.10	8.2	/	4.70	/	10	7.8	7	/	7.5	/	/
**PUFAs**	/	1.4	/	1.10	/	/	2.20	/	/	2.2	/	/	4.1	/	/
**MUFAs**	/	1.5	/	0.36	/	/	1.10	/	/	2.5	/	/	1.2	/	/
**SFAs**	1.1	1.3	/	1.41	1.1	/	1.40	/	0	3.1	3	/	2.2	/	/
**Carbohydrates**	15.1	11.3	18.9	20.42	27	/	60.70	/	23	6.9	29.7	/	31	/	/
**Sugar**	/	3.7	/	3.88	27	/	/	/	0	1.6	5	/	0	/	/
**Fiber**	7	16.4	/	3.50	13	/	13.00	/	0	16.1	<1	/	/	/	/
**Salt**	/	<0.1	/	2.12	3.7	/	/	/	0	<0.1	/	/	4.2	/	/
**Vitamins**	B_1_: 4.4 mg B_2_: 6.9 mg B_3_: 5.9 mg B_6_: 18.4 mg B_12_: 0.16 mg C: 1.4 mg D: 24 IU K: 1.3 mg	/	A: 68 mg C: 17 mg	A: 0.6 mg B_6_: 0.3 mg B_12_: 0.05 mg C: 5.1 mg K: 3.1 mg	B_12_: 350 µg	/	/	B_12_: 100 µg	Total vitamins: 60 µg	/	/	/	/	/	/
**Minerals and trace elements**	Ca: 270 mg Mg: 270 mg Fe: 82.7 mg K: 1040 mg Zn: 3.3 mg Na: 700 mg	/	Fe: 87 mg	Mg: 920 mg Mn: 4.6 mg Fe: 87 mg P: 102 mg Zn: 10.1 mg K: 1104 mg Cl: 112 mg F: 0.9 mg Cu: 1.84 mg I: 0.46 mg	Fe: 48 mg Ca: 130 mg	/	/	/	Fe: 102 mg	/	/	/	/	/	/
**Additional information**	Folic acid: 27 µg Chlorophyll: 1179 mg Carotenoids: 451 mg	/	/	/	/	/	Chlorophyll: 2.88 g	/	/	/	/	/	Ash: 15 g	/	/
**Origin**	/	China	China	China	/	/	/	Germany	/	China	China	China	Spain	/	/
**Expiration date**	10/2020	04/2021	07/2019	04/2020	05/2019	05/2020	04/2020	04/2019	07/2020	11/2020	06/2020	12/2019	04/2020	03/2020	01/2020
**Daily reference intake**	<5 g	3 Tea-spoons	3 g	5 g	5 g	4 g	5 g	3 g	4.5 g	3 Tea-spoons	2 g	1 Tea-spoon	0.25 g	2 g	0.012 g

AFA, *Aphanizomenon flos-aquae*; AP, *Arthrospira platensis*; CP, *Chlorella pyrenoidosa*; CV, *Chlorella Vulgaris*; DS, *Dunaliella salina*; HP, *Haematococcus Pluvialis*; TC, *Tetraselmis chuii*.

**Table 7:** Ranking of all 15 analyzed microalgae powders.

Ranking	Name	Positiv Characteristics	Negative Characteristics
1.	*Chlorella pyrenoidosa* No.3	↑↑AAS, ↑↑ protein, ↑↑ total fat, ↑↑↑ vitamin D_2_ + D_3_	↑ n6/n3 ratio
2.	*Chlorella vulgaris* No.2	↑↑↑ AAS, ↑↑ fiber, ↑↑ protein, ↑↑↑ chlorophyll, ↑↑↑ vitamin D_2,_ ↓↓↓ n6/n3 ratio	
3.	*Aphanizomenon flos-aquae*	↑↑ protein, ↑↑ Fe + Cu, ↑↑ vitamin D_3_ + δ-tocopherol, ↓↓↓ n6/n3 ratio	↑↑↑ C16:0
4.	*Chlorella pyrenoidosa* No.1	↑↑ AAS, ↑↑↑ fiber, ↑↑ minerals + Cu, ↓↓↓ heavy metals, ↑↑ vitamin D_2,_ ↓↓ n6/n3 ratio	
5.	*Chlorella vulgaris* No.1	↑↑ AAS, ↑↑ protein, ↑↑↑ fiber, ↑↑↑ chlorophyll, ↑↑ vitamin D_2_, ↓↓ n6/n3 ratio	
6.	*Tetraselmis chuii*	↑↑↑ carbohydrates, ↑↑ α-tocopherol, ↑↑ EPA, ↓↓↓ n6/n3 ratio	↑↑ As, ↓↓ chlorophylls
7.	*Chlorella pyrenoidosa* No.2	↑↑↑ fiber, ↑↑ vitamin D_2_, ↓↓ n6/n3 ratio	↓↓ trace elements, ↑↑ As
8.	*Arthrospira platensis* No.1	↑↑↑ AAS, ↑↑ protein, ↑↑ fiber, ↓↓↓ heavy metals	↓↓ minerals + trace elements, ↓↓↓ vitamins, ↑ n6/n3 ratio
9.	*Arthrospira platensis* No.5	↑↑ AAS, ↑↑ protein, ↑↑↑ fiber, ↑ Mg, Cu, Zn	↑ As, ↓↓↓ vitamins, ↑↑ n6/n3 ratio
10.	*Haematococcus pluvialis*	↑↑↑ carbohydrates, ↑↑↑ carotenoids, ↑↑ minerals, ↓↓ n6/n3 ratio	↓↓ total fat + protein, ↓↓ AAS, ↓↓↓ vitamins
11.	*Arthrospira platensis* No.3	↑↑↑ protein, ↑↑↑ minerals + Zn	↑↑↑ C16:0, ↑↑ n6/n3 ratio, ↓↓↓ vitamins
12.	*Arthrospira platensis* No.2	↑↑↑ protein, ↑↑↑ Zn	↑↑↑ C16:0, ↑↑ n6/n3 ratio, ↓↓↓ vitamins
13.	*Arthrospira platensis* No.4	↑↑↑ protein, ↑↑↑ Mo + Ca	↑↑↑ C16:0, ↑↑ n6/n3 ratio, ↑↑↑ As, ↓↓↓ vitamins
14.	*Dunaliella salina* No.2	↑↑↑ carbohydrates, ↑↑ Zn, ↑↑ α-tocopherol	↓↓↓ macronutrients, ↓↓↓ AAS, ↑ n6/n3 ratio
15.	*Dunaliella salina* No.1	↑↑↑ carbohydrates, ↑↑ Zn, ↑↑↑ α-tocopherol	↓↓↓ macronutrients, ↓↓↓ AAS, ↑ n6/n3 ratio, ↑↑ As+Pb

AAS, amino acid score; n3, omega-3-fatty acids; n6, omega-6-fatty acids; ↑ high, ↑↑ higher, ↑↑↑ very high; ↓ low, ↓↓ lower, ↓↓↓ very low.
